# Lack of the peroxiredoxin 6 gene causes impaired spatial memory and abnormal synaptic plasticity

**DOI:** 10.1186/s13041-021-00779-6

**Published:** 2021-04-19

**Authors:** Sarayut Phasuk, Sureka Jasmin, Tanita Pairojana, Hsueh-Kai Chang, Kai-Chi Liang, Ingrid Y. Liu

**Affiliations:** 1 Institute of Medical Sciences, Tzu Chi University, Hualien, Taiwan; 2 Department of Physiology, Faculty of Medicine Siriraj Hospital, Mahidol University, Bangkok, Thailand; 3 Department of Molecular Biology and Human Genetics, Tzu Chi University, Hualien, Taiwan; 4 Institute of Biomedical Sciences, Academia Sinica, Taipei, Taiwan

**Keywords:** Peroxiredoxin 6, Spatial memory, Long-term potentiation, Neuroinflammation, Reactive astrocyte

## Abstract

**Abstract:**

Peroxiredoxin 6 (PRDX6) is expressed dominantly in the astrocytes and exerts either neuroprotective or neurotoxic effects in the brain. Although PRDX6 can modulate several signaling cascades involving cognitive functions, its physiological role in spatial memory has not been investigated yet. This study aims to explore the function of the *Prdx6* gene in spatial memory formation and synaptic plasticity. We first tested *Prdx6*^*−/−*^ mice on a Morris water maze task and found that their memory performance was defective, along with reduced long-term potentiation (LTP) in CA3-CA1 hippocampal synapses recorded from hippocampal sections of home-caged mice. Surprisingly, after the probe test, these knockout mice exhibited elevated hippocampal LTP, higher phosphorylated ERK1/2 level, and decreased reactive astrocyte markers. We further reduced ERK1/2 phosphorylation by administering MEK inhibitor, U0126, into *Prdx6*^*−/−*^ mice before the probe test, which reversed their spatial memory deficit. This study is the first one to report the role of PRDX6 in spatial memory and synaptic plasticity. Our results revealed that PRDX6 is necessary for maintaining spatial memory by modulating ERK1/2 phosphorylation and astrocyte activation.

**Graphic Abstract:**

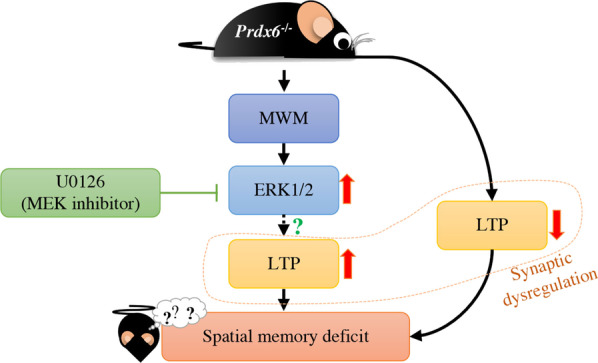

**Supplementary Information:**

The online version contains supplementary material available at 10.1186/s13041-021-00779-6.

## Highlights

- The *Prdx6* gene plays a critical role in spatial memory formation tested with the Morris water maze.
- Lack of *Prdx6* gene causes homeostatic dysregulation of hippocampal long-term potentiation (LTP).
- The *Prdx6*^−/−^ knockout mice have less reactive astrocytes in the hippocampus.
- Hyperphosphorylation of ERK1/2 in the hippocampus leads to impaired spatial memory of *Prdx6*^*−/−*^ mice.

## Introduction

Spatial memory impairment is one of the most common pathologies in aging processes, early Alzheimer’s disease, and some psychiatric disorders [1–3]. Oxidative damage and inflammation are two major causes of spatial memory impairment in these brain diseases [4]. The level of reactive oxygen species (ROS) is regulated by antioxidants during synaptic plasticity [5]. Either lower or higher levels of ROS than homeostatic level may cause impairment of synaptic plasticity and memory performance. Although growing evidence has revealed that various endogenous antioxidant enzymes regulate oxidative defense mechanisms and inflammatory responses [6, 7], their physiological roles in modulating cellular signaling for synaptic plasticity and spatial memory formation are not clear yet.

Peroxiredoxins (PRDXs) belong to a conserved 6-member superfamily of peroxidases that exist in many organisms [8]. Among the six members (PRDX 1–6), peroxiredoxin 6 (PRDX6) contains only 1-cysteine (1-cys) residue and is the only one that performs multiple functions, including peroxidase and acidic calcium-independent phospholipase A2 (aiPLA2) activities [8]. And it is expressed throughout the body, with a high expression level in the brain [9, 10]. The PRDX6 participates in oxidative defense mechanisms, phospholipid metabolism, lipid peroxidation repair, and inflammatory signaling [11–13]. It is also related to neurodegenerative diseases, including Parkinson's disease, Alzheimer's disease, and some diseases caused by chronic inflammatory response [14–16]. Gert Lubec et al. recently reported the relationship between antioxidant activity of PRDX6 and spatial memory performance [17]. Another study also revealed its role in inhibiting neurogenesis [10]. Since PRDX6 can either reduce or elevate ROS level, depending on the conditions [18–20], we hypothesized that PRDX6′s function might affect synaptic plasticity and spatial memory performance.

In the central nervous system (CNS), PRDX6 is dominantly expressed in the astrocytes, and not much in the neurons [21, 22]. The activation of astrocytes is required for maintaining sufficient energy supply to the neurons, the homeostasis of neurotransmitters, and the release of inflammatory cytokines such as tumor necrosis factor-alpha (TNFα) [23], interleukin 1 beta (IL-1β), and interleukin 6 (IL-6) [24]. Astrocytes are also crucial for modulating synaptic plasticity and memory performance [25, 26]. Molecular studies have revealed that PRDX6 can mediate several signaling molecules involving in memory processes, including ERK1/2 [27], protein kinase B (Akt) [28], p38 MAPK [27], as well as inflammatory cytokines like TNFα [15], IL-1β [15], IL-6 [29], and CC chemokine ligand 5 (CCL5) [30].

To identify the function of PRDX6 in spatial memory, we subjected *Prdx6* knockout (*Prdx6*^*−/−*^) mice to the MWM test, evaluated their motor coordination with the rotarod test, and measured their anxiety behavior with the light/dark transfer tests. Following the behavioral tests, we recorded hippocampal synaptic plasticity using the extracellular recording technique and measured expression levels of several related molecules in the hippocampus using western blot analysis.

## Materials and methods

### Animals

Dr. Shun-Ping Huang at Tzu Chi University, Taiwan provided *Prdx6* wild-type (*Prdx6*^+/+^) and knockout (*Prdx6*^−/−^) mice (12–14 weeks of age). The lack of the *Prdx6* expression was generated by replacing exons 1 and 2 with neomycin drug resistance and *Bgal* genes. The details for creating *Prdx6*^*−/−*^ mice were explained in the previous study [31]. Mutant mice were backcrossed with C57BL/6J mice for more than 9 generations. All mice were produced in our laboratory by mating a heterozygous knockout (*Prdx6*^+/−^) male with two *Prdx6*^+/−^ female mice or intercrossed with the same genotypes. The genotypes were confirmed by polymerase chain reaction (PCR) using specific primers (Additional file 2: Table. S1) [31]. Mice were maintained in the Laboratory animal center of Tzu Chi University with ad libitum access to food and water under a constant 12 h light/dark cycle. All procedures were reviewed and approved by the Institutional Animal Care and Use Committee of Tzu Chi University, Taiwan (approval #104099), and is in accordance with the Taiwan Ministry of Science and Technology guidelines for the ethical treatment of animals.

### Intraperitoneal injection (i.p.) of MEK inhibitor (U0126)

The MEK inhibitor U0126 was purchased from Promega (#V112A, Promega Co., USA). The inhibitor was then dissolved in 234 μl of DMSO to obtain stock concentration (10 mM). The inhibitor was then diluted in 0.9% normal saline to get the final concentration 50 μM for further experiment. We used normal saline containing 0.5% DMSO as the sham control solution (vehicle). In the present study, we used intraperitoneal injection to deliver the inhibitor U0126 [32]. Mice were intraperitoneally injected with 100 μl of 0.9% normal saline every day from the first day of the visible platform trial until the last day of training to exclude the effect of handling and injection. One hour before the probe test, mice received 100 μl of either vehicle or U0126 via i.p. injection. The protein samples were then collected from the hippocampi immediately after the completion of the probe test.

### Behavioral experiments

#### Morris water maze test

**Fig. 1:**
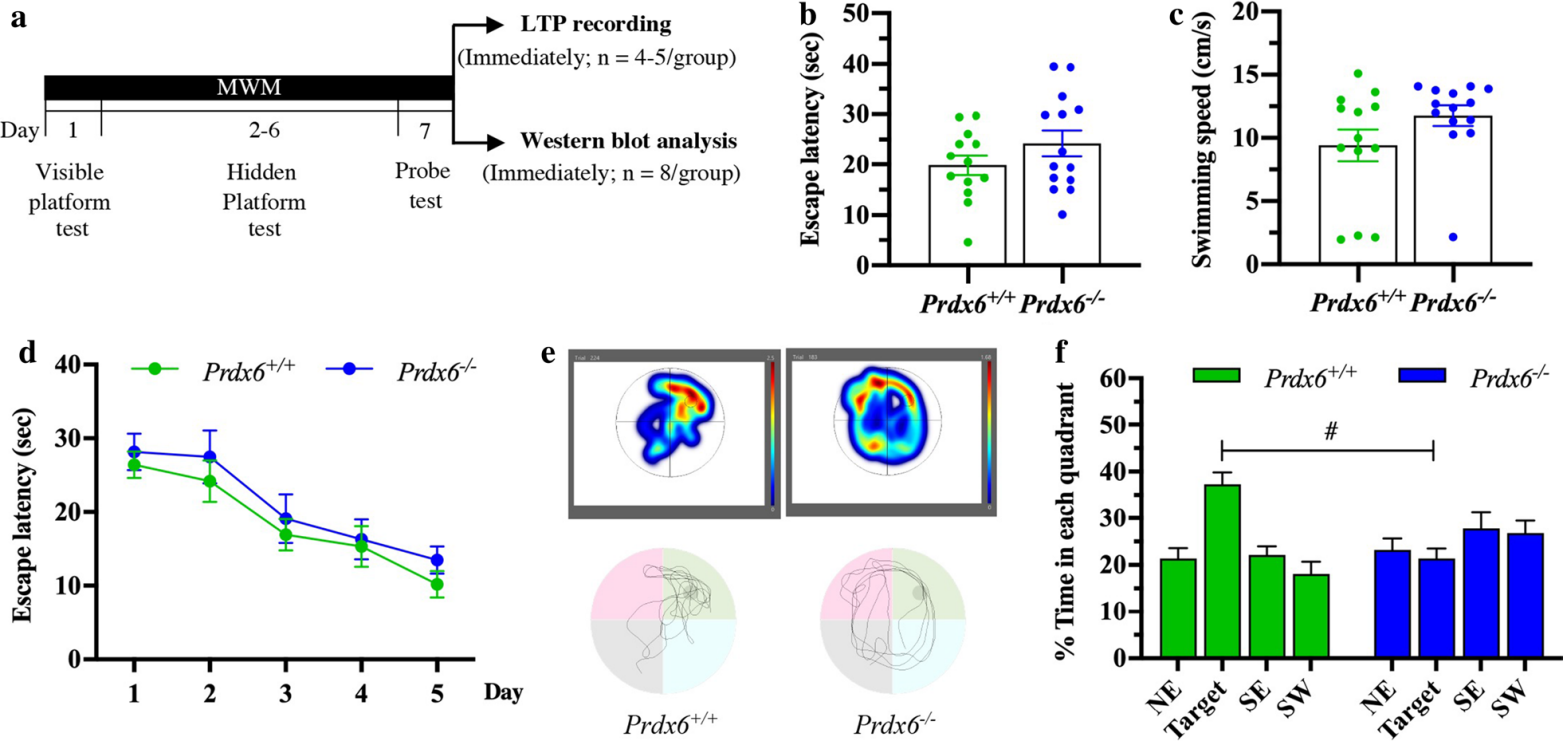
*Prdx6*^*−/−*^ mice displayed impaired performance of spatial reference memory in the Morris water maze (MWM) task. **a** Timeline of MWM task and sample collection. **b** Escape latency to platform, and **c** mean swimming velocity during visible platform trial (n = 13–14 mice/group). **d** Learning curve during 5 days of training. **e** Representation of the time spent throughout the water maze using a heat map (upper panel) and illustration of the swimming pattern (lower panel) during the probe test. **f** Percent time spent in each quadrant during probe test. All data are presented as mean ± SEM. ^#^*p* < 0.05, mixed-design repeated measure ANOVA followed by Bonferroni’s post hoc test with unpaired Student’s *t* test for individual differences between groups within each quadrant

The procedure for Morris water maze test was adapted from the previous study [33]. A circular pool (diameter 110 cm, height of the platform—21 cm) was filled with water at room temperature (21 °C ± 1 °C). The water was made opaque with a non-toxic white paint (Cat. # 187203, Palmer paint products, USA). Four points equally dividing the pool into four quadrants, and a round platform (10 cm in diameter) was placed in one of the quadrants. The visible platform test was carried out on the first day (6 trials per day). We placed the platform 0.5 cm above the water surface and trained the mice to find the visible platform within 60 s. The starting point for each trial was randomly selected among the four quadrants (Additional file 2: Table. S2). In the hidden platform test, the platform was kept 1 cm beneath the water surface in the northeast (NE) quadrant. Mice were randomly placed into the water maze but not in the southwest quadrant (SW) (Additional file 2: Table. S3). They were given six trials per day, each for 60 s, to find the hidden platform for five consecutive days. If they did not reach the platform, they were guided to the platform and left there for 10 s for the mice to locate the platform with visual cues. On day 7, a probe test was performed with the platform removed from the pool. Each mouse was placed at an unfamiliar starting point (southwest, SW) and allowed to swim freely for 60 s. We used a video camera and tracking system (EthoVision XT 15, Noldus Information Technology) to measure the escape latency, swimming speed, and percentage of time spent in each quadrant. After completing a probe test, we collected 8 protein samples from 8 mice and used the remaining mice for LTP recording (Fig. 1a). The corresponding behavioral data was shown in Fig. 1. In this study, the same animals were used for Figs. 1, 4, 5, and 7.

#### Open field test

**Fig. 2:**
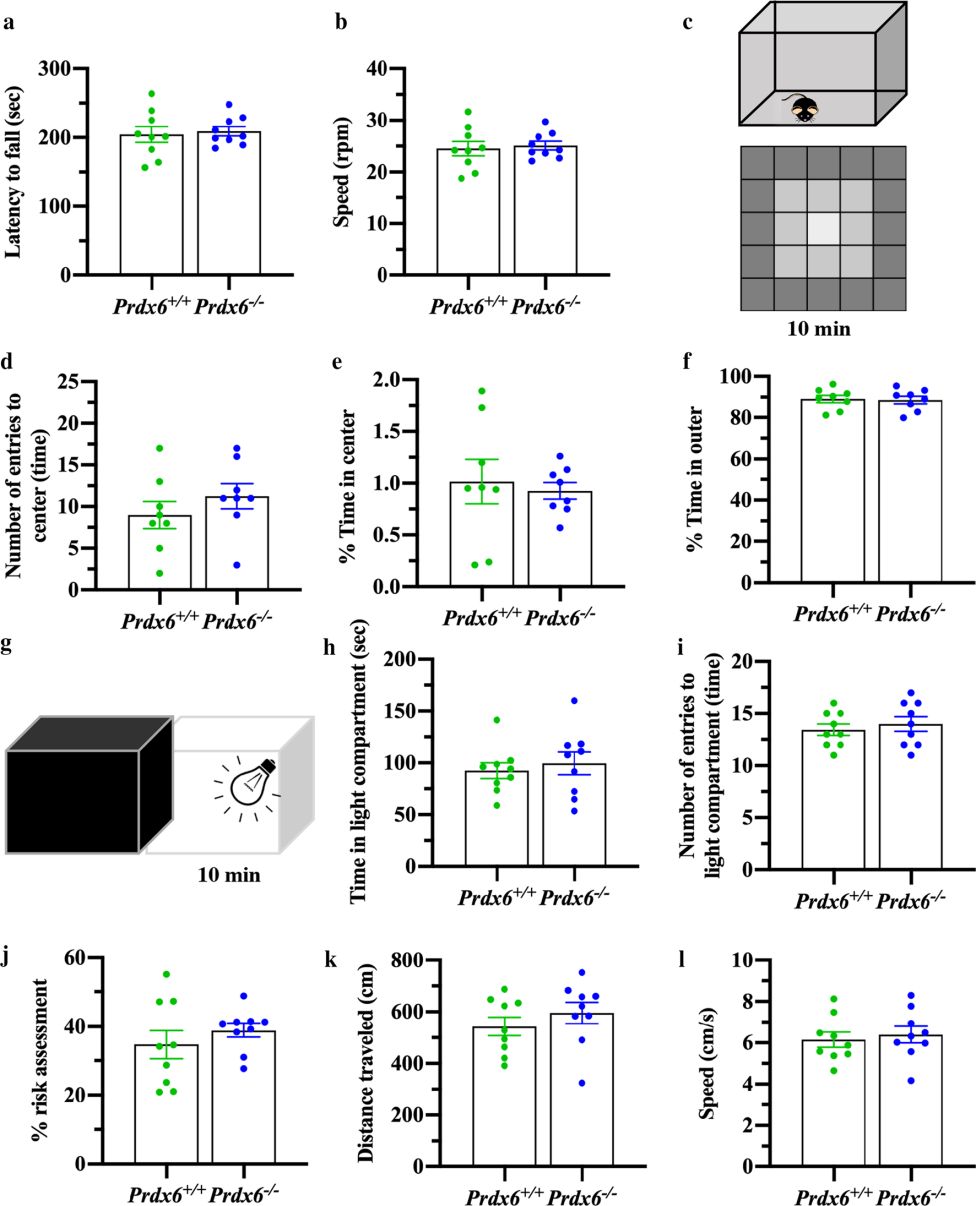
Normal locomotor functions and anxiety-like behaviors in *Prdx6*^*−/−*^ mice. **a** Time remained on an acceleration rota rod (sec) before falling (n = 9 mice/group) and **b** mean rotational velocity (rpm) at the time of falling. **c** Schematic of zones in the open field arena. **d** Number of entries into the center of an open field arena (n = 8 mice/group). **e** and **f** Percent time spent in the center (**e**) and outer (**f**) areas in the open field arena. **g** Schematic of light/dark transfer test. **h** Time spent in the light compartment (n = 9 mice /group). **i** Number of entries into the light compartment. **j** Percent risk assessment during 10 min of exploration. **k** Distance traveled, and **l** moving speed in the light compartment during light/dark transfer test. All data are presented as mean ± SEM., unpaired Student’s *t* test following a normal distribution

To investigate their motor function and anxiety response, another batch of animals was used for Fig. 2. The open field chamber was placed under the dim light condition with a top-down video recorder. Mice were allowed to explore the nontransparent chamber for 10 min freely. The distance traveling and moving speed were analyzed by video tracking software (EthoVision XT 15, Noldus Information Technology) and were calculated to measure locomotor activity. For anxiety-like behavior, the chamber was divided into three areas (outer, middle, and inner) to obtain the time spent in each area along 10 min of exploration.

#### Rotarod test

For the rotarod test, mice were left on the rotating rod for 60 s per trial with constant speed on the training day. To evaluate the motor coordination, trained mice were set on the rod with a gradual increase in speed from 4 to 40 rpm within 300 s per trial. The latency to fall and maximum speed was calculated.

#### Light–dark transfer test

The light–dark apparatus is divided into two compartments, dark (25 × 25 × 35 cm) and light (25 × 25 × 35 cm, 700 lx) connected with the sliding door located on the floor at the center of the partition. The mice were placed in a light division and allowed to explore the apparatus for 5 min freely. The distance traveled and moving speed were recorded using a camera, and scored using automated software (EthoVision XT 15, Noldus Information Technology). To investigate the anxiety-like behavior, total time spent in the light compartment and number of entries to the light compartment were recorded. Time spent in the risk arena (3 cm length-wise × 6 cm width-wise surrounding the sliding door) and in the outside risk assessment zone was also calculated as the percentage of risk assessment time.

### Extracellular LTP recording

We adapted the procedure reported in a previous study with a slight modification to record long-term potentiation [34]. Both *Prdx6*^+*/*+^ and *Prdx6*^*−/−*^ mice were anesthetized with isoflurane and sacrificed by guillotine at naïve (basal) condition, and after the completion of the probe test. Whole brains were immediately removed and washed in ice-cold artificial cerebrospinal fluid (ACSF). The brains were then sticked on the metal chamber of a vibrating microtome (Leica VT1000 S, Leica Biosystems Inc., Nussloch, Germany). The brains were horizontally sectioned in oxygenated (95% O_2_ and 5% CO_2_) ACSF at 350 μm thickness. The brain slices were transferred into the tissue chamber containing oxygenated ACSF in the incubated boxes and recovered at temperature 28–30 °C for at least 2 h. The brain slices were transferred to a recording chamber for extracellular LTP recording. During recording, hippocampal slices were perfused with oxygen (O_2_) saturated ACSF at a speed of 2–3 mL/min with 28 °C. The glass pipettes were pulled on a micropipette puller (PC-10 Needle Puller, Narishige, Japan) filled with normal ACSF. This recording electrode was placed at the stratum radiatum of the CA1 region for recording the excitatory postsynaptic field potentials (fEPSPs). The unipolar stainless-steel microelectrodes (Frederick Haer Company, Bowdoinham, ME, USA) were used as a stimulus electrode. The stimulation intensity was adjusted between 4 and 15 V for each slice, so that the fEPSP were elicited to approximately 35–40% of the maximal response. Baseline fEPSP were evoked every 20 s for 10 and 20 min followed by high-frequency stimulation (HFS), which includes 3 trains of 100 pulses at 100 Hz for 60 s. Then, fEPSPs were stimulated every 20 s for an additional 60 min or 3 h. Recordings were amplified using an Axon Multiclamp 700B amplifier (Axon Instruments, Foster City, CA). All signals were filtered at 1 kHz and digitized at 10 kHz by Axon Digidata 1550B plus HumSilencer (Axon Instruments, Foster City, CA) using Signal software. The downward slope of fEPSPs was recorded and analyzed by Axon pCLAMP 11 software.

### Western blot analysis

After the completion of the probe trial, mice were immediately anesthetized and decapitated to extract proteins from the brain. The whole hippocampi were isolated and collected in ice-cold RIPA lysis buffer 1 × (Millipore, USA) containing protease and phosphatase inhibitor. The homogenized tissues were kept on ice for another 30 min before centrifuging at 13,000 rpm for 15 min at 4 °C. The supernatants were transferred to new Eppendorf tubes and stored at − 80 °C for further experiments. The protein samples (30–50 μg of protein) were loaded and run on 10% SDS-PAGE at 80 V in stacking gel and 140 V in resolving gel. The separated proteins were transferred to a PVDF membrane (0.4 μm pore size) at 30 V in a cool room overnight. The blots were incubated with anti-BDNF (1:2000; Abcam, UK), anti-PSD95 (1:2000; ThermoFisher, USA), anti-pERK1/2 (1:2000; Cell Signaling, Danvers, MA), anti-pAkt1 (1:1000; Cell signaling Technology, USA), anti-pCaMKII (1:2000; Abcam, UK), anti-GFAP (1:2000; Abcam, UK), anti-TNF-α (1:1000; Abcam, UK), anti-IL6 (1:2000; Proteintech, USA) or anti-β-actin antibody (1:10,000; Sigma-aldrich) in TBST containing 0.1% BSA (ThermoFisher, USA) overnight at 4 °C room on a shaker. The blots were then incubated with either horseradish peroxidase-conjugated secondary antibody goat anti-mouse IgG (Cell signaling, Danvers, MA) or goat anti-rabbit (Santa Cruz Biotechnology, Santa Cruz, CA, USA) diluted 1:5000 or 1:10,000 in 0.1% BSA in TBST for 1 h at room temperature. The detail information for the antibodies used in this study is provided in Additional file 2: Table S4. The blots were washed 10 min for three times with TBST before imaging. For visualization, the blots were developed using ECL (Western lighting® Plus ECL, PerkinElmer Inc, MA, USA) and detected under (Bio Rad ChemiDoc MP High performance Cold light Fluorescence Analysis system). The band intensities were quantified using ImageJ 1.52a (National Institutes of Health, USA).

### Statistics

The sample sizes of the animals for each experiment were provided in an Additional File 2: Table S5. Statistical analysis was performed using SPSS (version 25, IBM Corporation), and the graphs were plotted using GraphPad Prism version 8. After assessing the normality using the Shapiro–Wilk test, Student's *t*-tests were conducted compared to two independent groups with a normal distribution. In contrast, data without normal distribution was examined with Mann–Whitney U-test. For learning ability of hidden platform trials and time spent in each quadrant during probe test, the results were analyzed as mixed-design repeated-measures ANOVA with trials as within-subjects factor and genotypes as a between-subjects factor. We used a Bonferroni-corrected *t*-test (adjusted *p* ≤ 0.025 for 2 statistical tests and *p* ≤ 0.017 for 3 statistical tests) to examine statistical differences between two independent groups.

For the MEK inhibitor experiment, escape latency and swimming speed were analyzed by two-way measure ANOVA with genotype and intervention as independent factors followed by Bonferroni's post hoc test. All data are presented as mean ± SEM, with statistical significance at *p* < 0.05. Sample sizes are described in figure legends.

## Results

### *Prdx6*^−/−^ mice exhibited impaired spatial memory

To examine hippocampal-dependent spatial memory in *Prdx6*^−/−^ mice, we first conducted the Morris water maze (MWM) test (Fig. 1a). To ensure that the mice have no problem with their visual ability and locomotor function, we performed a visible platform trial on day 1 of the test. In a visible platform trial, the statistical analysis using unpaired Student’s *t*-test demonstrated that there was no significant difference in the time to reach the visible platform (*t*_25_ = − 1.330, *p* = 0.196, Fig. 1b) and in the mean speed of swimming (*t*_25_ = − 1.594, *p* = 0.123, Fig. 1c) between the two genotypes. It suggested that *Prdx6*^−/−^ mice have normal visual and sensorimotor function.

The hidden platform was fixed in the northeast (NE) quadrant in acquisition trials as a target quadrant. And the mice were then randomly placed at different starting points. Using mixed-design ANOVA, there was no effect of genotype on escape latency to find a hidden platform (*F*_(1,25)_ = 0.674, *p* = 0.420, Fig. 1d). Both groups spent equal time locating the hidden platform across the five days of training. There was no significant effect of the interaction between the genotype and training day on escape latency (*F*_(4,100)_ = 0.136, *p* = 0.969, Fig. 1d). Mice of both genotypes exhibited a normal ability to find the hidden platform indicating in decreased escape latency from training day 1 to 5, as shown by the main effect of the training day (*F*_(4,100)_ = 23.487, *p* = 0.000, Fig. 1d). Further unpaired Student's *t*-testing revealed no difference between the two genotypes (*p* > 0.05, Fig. 1d). These results suggested that deficiency of PRDX6 does not affect the acquisition of spatial memory.

To confirm that the mice used their spatial memory to find the hidden platform, mice were allowed to swim in the maze for a total of 60 s without the platform during the probe trial. The heat map (upper panel) and swimming patterns (lower panel) during the probe test are illustrated in Fig. 1e. We found that *Prdx6*^+*/*+^ mice displayed statistical differences in percent time spent in the four quadrants (*F*_(3,36)_ = 10.309, *p* = 0.000, Fig. 1f), with the longest time in the target quadrant (NE) (All Bonferroni's post hoc tests were significant, *p* < 0.05). On the other hand, *Prdx6*^*−/−*^ mice spent equal time in all quadrants (*F*_(1.954,25.406)_ = 0.894, *p* = 0.0419, Fig. 1f). Using Bonferroni-corrected *t*-test analysis, we observed that *Prdx6*^−/−^ mice spent significantly less time in the target quadrant than *Prdx6*^+/+^ mice (*t*_25_ = 4.814, *p* = 0.000, Fig. 1f). These data proved that *Prdx6*^*−/−*^ mice exhibited spatial memory impairment in the MWM test.

### Spatial memory deficit in *Prdx6*^−/−^ mice was not due to locomotor activity, motor coordination, and anxiety level

We next evaluated the motor function and anxiety response of these knockout mice. Results showed increase in distance traveled (*t*_-2.749_ = 14.000, *p* = 0.016, Additional file 1: Fig. S1b) and moving speed (*t*_-2.741_ = 14.000, *p* = 0.016, Additional file 1: Fig. S1b) of *Prdx6*^*−/−*^ mice. These results indicated that the locomotor function of *Prdx6*^*−/−*^ mice is comparable to that of their wild-type littermates. To verify the influence of motor coordination in spatial memory impairment, a rotarod test was performed on another batch of animals to avoid any confounding factor. There was no difference between the two genotypes on latency to fall (*t*_16_ = − 0.356, *p* = 0.726, Fig. 2a) and speed (*t*_16_ = − 0.356, *p* = 0.726, Fig. 2b). These results indicated that *Prdx6*^*−/−*^ mice's spatial memory deficit is not affected by locomotor dysfunction.

The ability to navigate using spatial cues or swim in a water maze can interfere with anxiety levels [35]. To verify the influence of anxiety level on spatial memory impairment of *Prdx6*^*−/−*^ mice, we subjected the mice to an open field (Fig. 2c) and light/dark transfer test (Fig. 2g). After assessing the normality by using the Shapiro–Wilk test, we observed no significant difference in the number of entries to center (*t*_14_ = − 1.011, *p* = 0.329, Fig. 2d), percentage time spent in center (*t*_14_ = 0.387, *p* = 0.705, Fig. 2e), and percentage time spent in the outer zone (*t*_14_ = 0.203, *p* = 0.842, Fig. 2f) between the two genotypes. In the light/dark transfer test, no significant difference was recorded in time spent in (*t*_16_ = − 0.538, *p* = 0.598, Fig. 2h) and number of entries into (*t*_16_ = − 0.618, *p* = 0.545, Fig. 2i) the light compartment between the two genotypes. When the risk area was drawn in the light compartment, *Prdx6*^*−/−*^ mice have the same percentage of risk assessment compared to their wild-type mice (*t* = − 0.894, *p* = 0.385, Fig. 2j). We also observed equal distance traveled (*t*_16_ = − 0.945, *p* = 0.359, Fig. 2k) and moving speed (*t*_16_ = − 0.450, *p* = 0.659, Fig. 2l) of *Prdx6*^*−/−*^ mice when compared to *Prdx6*^+/+^ mice. The results obtained from the open field and light/dark transition tests confirmed that spatial memory impairment of *Prdx6*^*−/−*^ mice was not affected by their anxiety-like behavior.

### Electrophysiological recording of hippocampal slices sectioned from home-caged *Prdx6*^*−/−*^ mice showed reduced hippocampal long-term potentiation (LTP) 3 h after HFS

**Fig. 3:**
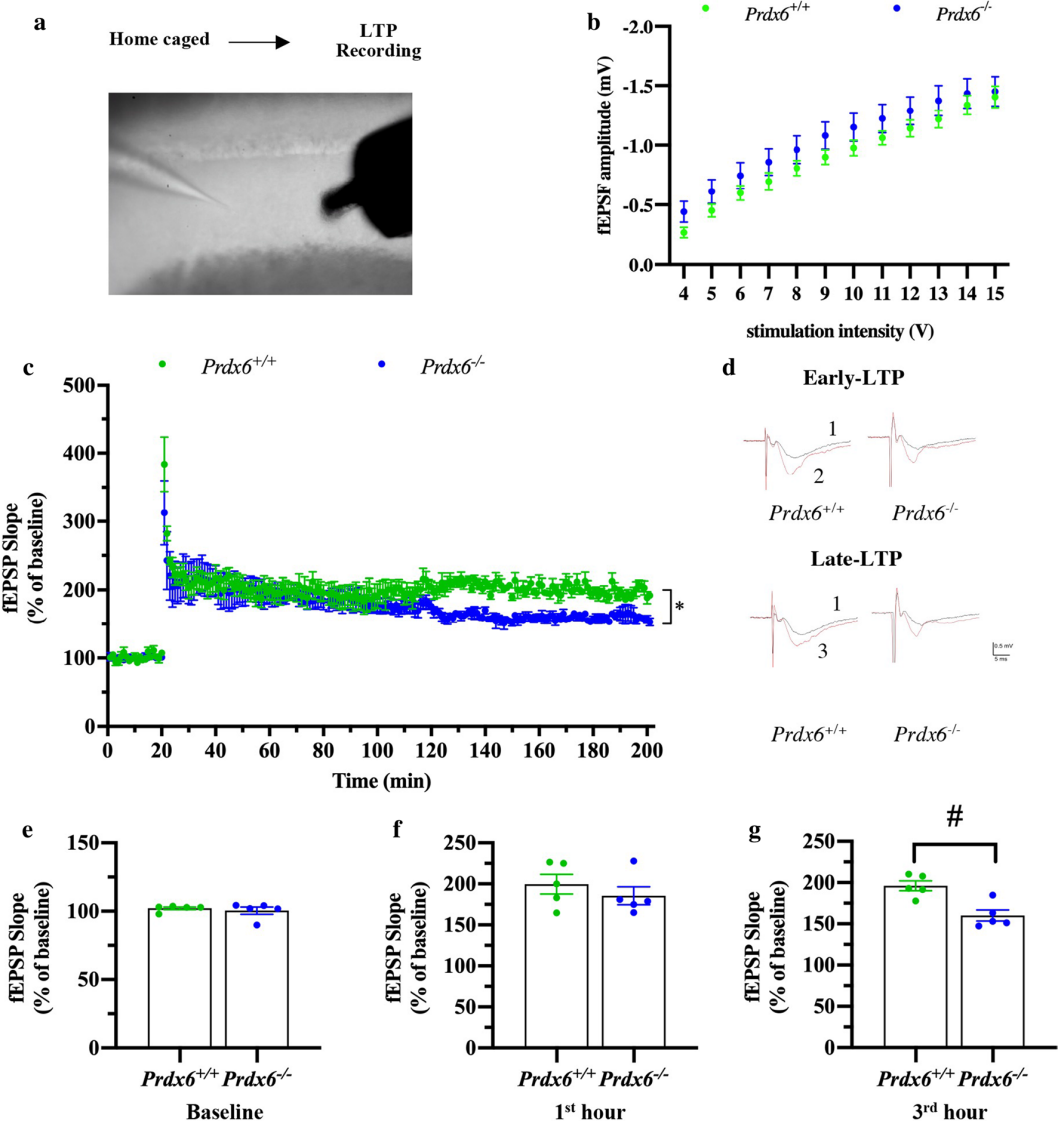
Decline of hippocampal LTP in *Prdx6*^*−/−*^ mice recorded from basal condition. **a** Schematic representation of LTP depicting stimulation and recording in hippocampal CA1-CA3 synapse. **b** Input–output curve of fEPSP slope (n = 8 slices/5 mice in each group). **c** Average fEPSP plotted against time in minutes (n = 5 mice/group). **d** Example traces representing the average of 3 sweeps. **e**–**g** The average of fEPSP slopes from the last 10 min of the first hour and third hour after HFS of *Prdx6*^+*/*+^ and *Prdx6*^*−/−*^ mice were calculated and plotted. Normalized fEPSP slope (%) for baseline (**e**), first hour (**f**) and third hour (**g**) of hippocampal LTP. All data are presented as mean ± SEM. ^#^*p* < 0.05. Unpaired Student’s *t* test following a normal distribution and mixed-design repeated measure ANOVA followed by Bonferroni’s post hoc test with unpaired Student’s *t* test for individual differences between groups within training day

To understand the cellular mechanism underlying the impairment of spatial memory in *Prdx6*^*−/−*^ mice, we then conducted LTP recording in the CA1 region of acute hippocampal slices taken from home-caged mice (Fig. 3a). No difference in overall input–output curves (*F*_(1,14)_ = 1.455, *p* = 0.248, Fig. 3b) between the two genotypes was recorded. Time course of LTP recorded from the CA1 region of *Prdx6*^*−/−*^ and *Prdx6*^+/+^ mice and the average of 3 traces recorded during baseline, 1st hour, and 3rd hour of LTP were shown in Fig. 3c and d, respectively. Non-parametric test indicated no statistical difference of the average percentage of baseline fEPSP slope evoked in Schaffer collateral stimulation between the two genotypes (*U* = 12, *p* = 0.917, Fig. 3c, e). The enhancement of LTP slope averaged throughout 3 h after high-frequency stimulation (HFS) was observed in both *Prdx6*^+*/*+^ and *Prdx6*^*−/−*^ mice (*F*_(1,8)_ = 221.436, *p* = 0.000, Fig. 3c). Slope of LTP was similar (last 10 min of first hour after HFS) (*t*_8_ = 0.879, *p* = 0.405, Fig. 3f) between the two genotypes. LTP slope started from 2 h after HFS was significantly decreased and maintained at a lower level during 120–180 min (*t*_8_ = 4.005, *p* = 0.004, Fig. 3g) in hippocampal slices prepared from *Prdx6*^*−/−*^ mice. These results indicated that lack of the *Prdx6* causes the LTP reduction in the hippocampal CA1 region.

### Enhanced CA3-CA1 hippocampal long-term potentiation (LTP) was recorded in *Prdx6*^*−/−*^ mice after the probe test

**Fig. 4:**
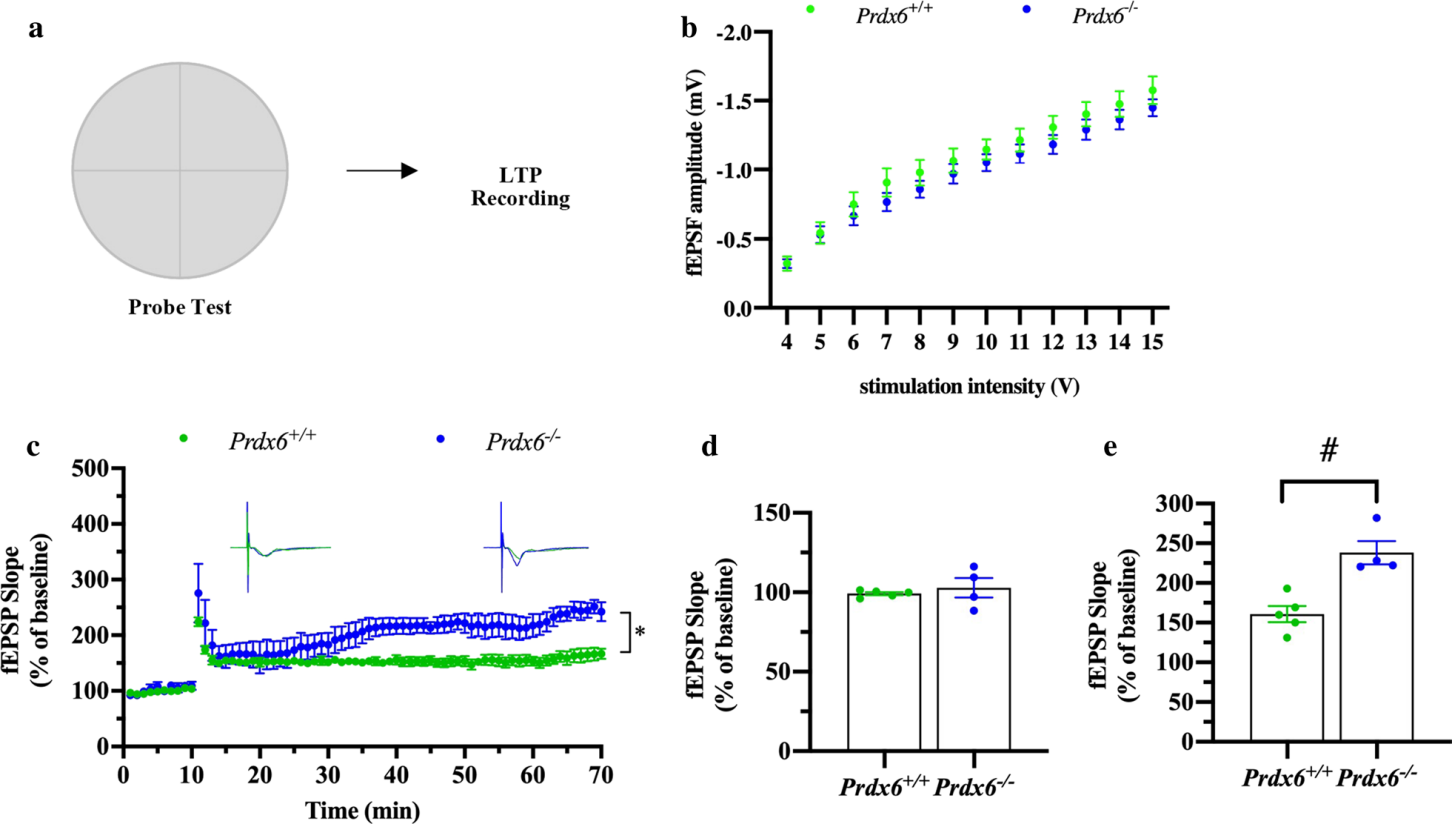
Enhanced hippocampal LTP in *Prdx6*^*−/−*^ mice after probe test. **a** Schematic representation of LTP depicting stimulation and recording in hippocampal CA1-CA3 synapse after the completion of probe test. **b** Input–output curve of fEPSP slope (n = 8 slices/4–5 mice in each group). **c** Average fEPSP plotted against time in minutes (n = 4–5 mice/group). **d** and **e** Normalized fEPSP slope (%) for baseline (**d**) and first hour (**e**) of hippocampal LTP. All data are presented as mean ± SEM. ^#^*p* < 0.05, unpaired Student’s *t*-test following a normal distribution

To understand whether the lack of the *Prdx6* gene affects hippocampal plasticity during memory retrieval, the same set of animals described in Fig. 1a were subjected to the MWM test. After the completion of the probe test, the brains were immediately removed and sectioned for extracellular recording (Fig. 4a). Field recording of fEPSPs in CA1 of the hippocampus showed no significant difference between *Prdx6*^*−/−*^ and *Prdx6*^+/+^ mice in input–output curves (*F*_(1,12)_ = 0.993, *p* = 0.339, Fig. 4b). In this experiment, we recorded LTP for 60 min (Fig. 4c). The baseline fEPSPs evoked in Schaffer collateral stimulation are similar in both groups (*t*_7_ = − 0.653, *p* = 0.535, Fig. 4c, d). The slope of fEPSPs along 1 h of recording indicated that high-frequency stimulation (HFS; 100 Hz for 1 s, 3 trains) induced the induction of hippocampal long-term potentiation (LTP) in both *Prdx6*^*−/−*^ and *Prdx6*^+/+^ mice (*F*_(1,7)_ = 175.403, *p* = 0.000, Fig. 4c). Interestingly, we observed enhanced fEPSP slope calculated at the last 10 min (*t*_7_ = − 4.447, *p* = 0.003, Fig. 4e) in *Prdx6*^*−/−*^ mice compared to *Prdx6*^+*/*+^ mice.

### Hyperphosphorylation of ERK1/2 and upregulation of PSD95 and cPLA2 were detected in the hippocampus after a probe test in *Prdx6*^−/−^ mice

**Fig. 5:**
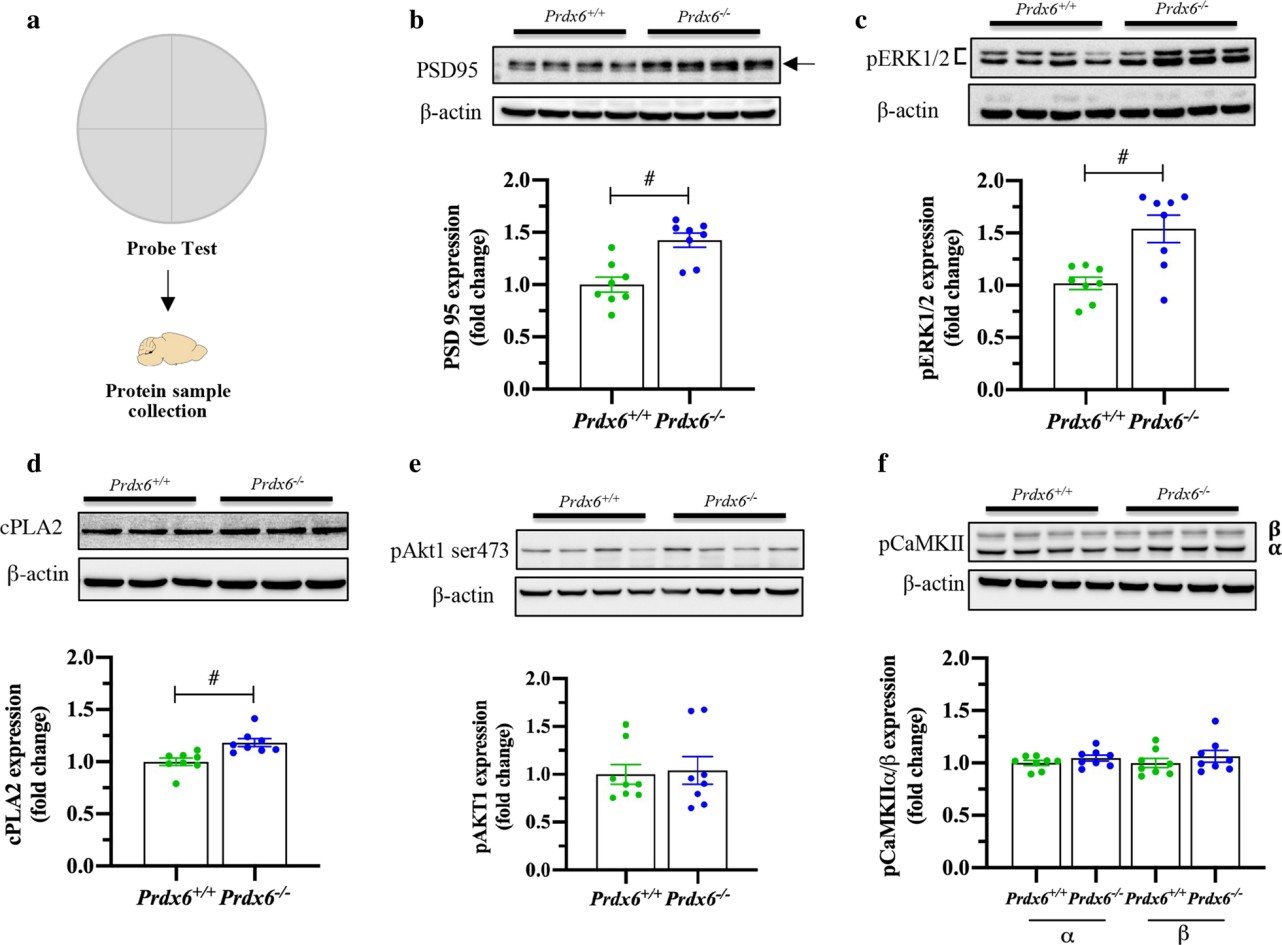
*Prdx6*^*−/−*^ mice showed an increase in MAPK signaling in the hippocampus at the time of memory retrieval. **a** Schematic of total protein collection from the hippocampus immediately after the probe test. **b**–**f** Representative western blot and quantification of PSD95 (**b**), pERK1/2 (**c**), cPLA2 (**d**), pAkt1 ser473 (**e**), and pCaMKIIα/β (**f**) in the hippocampi collected immediately after the probe test (n = 8 mice/group). All data are presented as mean ± SEM. ^#^*p* < 0.05, unpaired Student’s *t*-test following a normal distribution and Mann-Whitney U test following a non normally distributed

To better understand the molecular mechanism underlying spatial memory deficit, we investigated expression of various synaptic proteins after the probe test (Fig. 5a), including BDNF, PSD95, phosphorylated ERK1/2, cPLA2, phosphorylated Akt1, and phosphorylated CaMKII. Western blot analysis revealed no significant difference in pro BDNF (*t*_14_ = 0.378, *p* = 0.711, Additional file 1: Fig. S2a, b) and mature BDNF (*t*_12_ = − 1.021, *p* = 0.328, Additional file 1: Fig. S2a, c) between the two genotypes. On the other hand, we detected a significant increase in PSD95 (*t*_14_ = − 4.269, *p* = 0.001, Fig. 5b), pERK1/2 (*t*_14_ = − 3.640, *p* = 0.003, Fig. 5c), and cPLA2 (*t*_13_ = − 3.607, *p* = 0.003, Fig. 5d) level in the hippocampus of *Prdx6*^*−/−*^ mice compared to *Prdx6*^+*/*+^ mice. Since AKT1 and CaMKII are a promising target of BDNF/TrkB signaling [36], we thus examined the levels of pAkt1 and pCaMKII. Statistical analysis revealed no significant differences of pAkt1 (*U* = 29, *p* = 0.753, Fig. e), pCaMKIIα (*t*_14_ = − 1.262, *p* = 0.228, Fig. 5f) and pCaMKIIβ (*t*_14_ = − 0.892, *p* = 0.387, Fig. 5f) in the hippocampi of *Prdx6*^*−/−*^ mice. These results indicated that phosphorylation of ERK signaling pathway is associated with enhancement of hippocampal LTP after probe test in *Prdx6*^*−/−*^ mice.

### Administration of MEK inhibitor, U0126, before the probe test prevents spatial memory decline of *Prdx6*^−/−^ mice

**Fig. 6:**
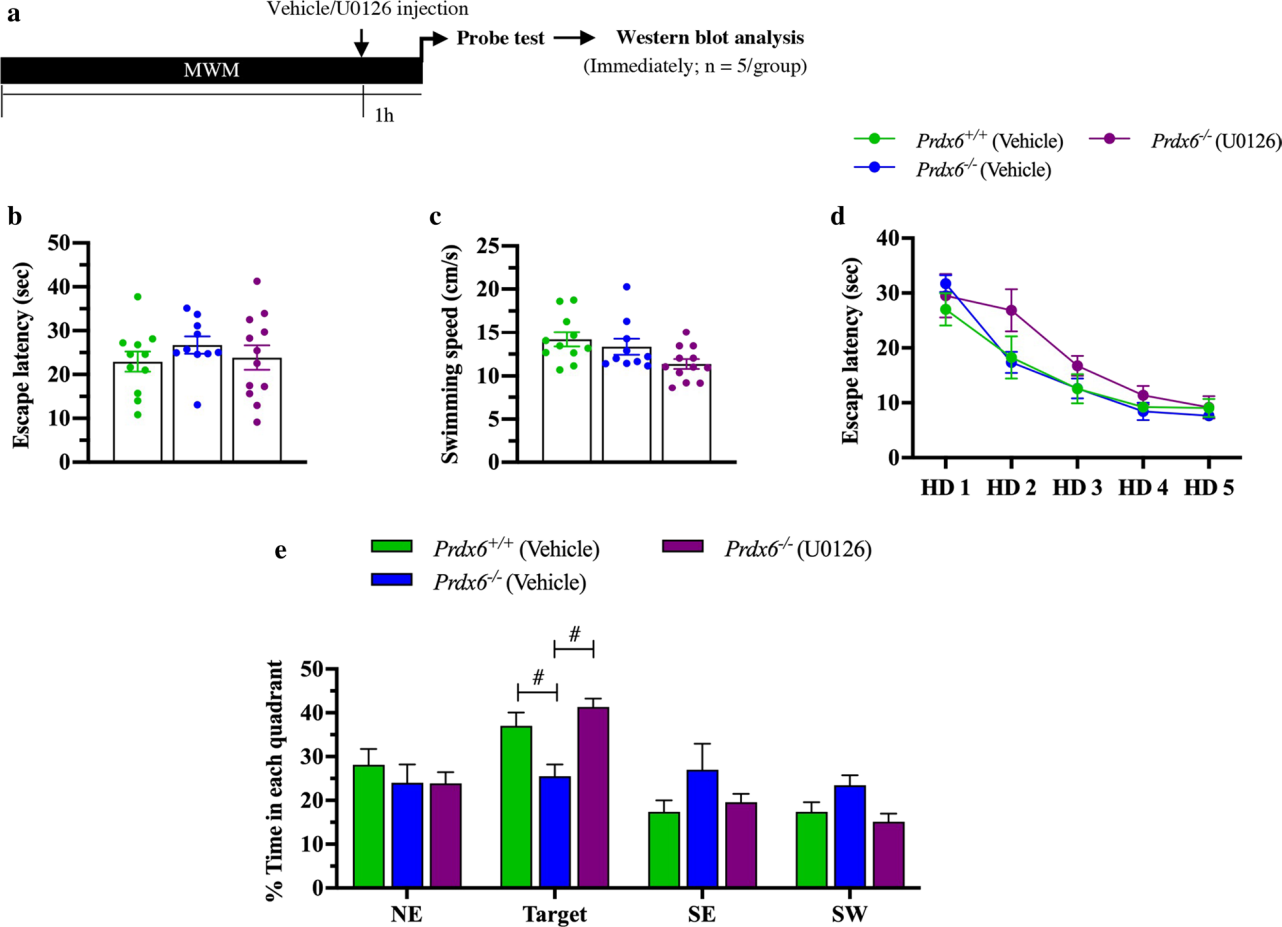
Reducing phosphorylated ERK1/2 level rescued spatial memory impairment of *Prdx6*^*−/−*^ mice. **a** MEK inhibitor (U0126) injection before probe test. **b** Escape latency to platform and **c** mean swimming velocity during visible platform trial (n = 10–12 mice/group). **d** Learning curve during 5 days of training. **e** Percent time spent in each quadrant during the probe test. All data are presented as mean ± SEM. ^#^*p* < 0.05, two-way measure ANOVA followed by Bonferroni’s post hoc test with one-way ANOVA for multiple comparison of percent time spent in target quadrant

To confirm whether excessive ERK1/2 phosphorylation is the cause of memory deficit found in *Prdx6*^−/−^ mice, we injected MEK inhibitor (U0126) 1 h before the probe test (Fig. 6a) and examined mice’s performance. One-way ANOVA analysis revealed that there was no significant effect of genotype on the time for reaching the visible platform (*F*_(1,28)_ = 1.135, *p* = 0.296, Fig. 6b), and the mean of swimming speed (*F*_(1,28)_ = 0.579, *p* = 0.453, Fig. 6c). These results suggested that *Prdx6*^−/−^ mice have a normal sensorimotor function. In acquisition trials, using mixed-design ANOVA, there were no effects of the group as well as the interaction between the group and training day on escape latency to find a hidden platform (*F*_(2,28)_ = 1.371, *p* = 0.270 and *F*_(5.85,81.907)_ = 1.394, *p* = 0.228, respectively, Fig. 6d). Both genotypes exhibited a normal ability to find the hidden platform, indicated by decreased escape latency from training day 1 to 5 as shown by the main effect of the training day (*F*_(2.925,81.907)_ = 55.537, *p* = 0.000, Fig. 6d). In probe test, using Bonferroni-corrected *t*-test analysis with adjusted *p* value less than 0.0167 served as a statistically significant difference, we observed that *Prdx6*^−/−^ (vehicle) spent significantly less time in the target quadrant than *Prdx6*^+/+^ (vehicle) group (*t*_19_ = 2.790, *p* = 0.012, Fig. 6e). Interestingly, administration of U0126 significantly improved spatial memory of *Prdx6*^−/−^ mice compared with *Prdx6*^+/+^ mice without inhibitors (*t*_19_ = − 5.117, *p* = 0.000, Fig. 6e). Besides, compared with their wild-type littermates, *Prdx6*^*−/−*^ mice with U0126 administration spent comparable time in the target quadrant (*t*_20_ = − 1.354, *p* = 0.191, Fig. 6e). We also confirmed a significant decrease in pERK1/2 (*F*_(2,14)_ = 61.776, *p* = 0.000, Additional file 1: Fig. S3a) in the hippocampi of *Prdx6*^*−/−*^ mice treated with U0126 when compared to vehicle treated knockout mice. These results proved that hyperphosphorylated ERK1/2 in the hippocampus causes spatial memory impairment in *Prdx6*^*−/−*^ mice. Therefore, reducing ERK1/2 phosphorylation before the probe test rescues *Prdx6*^*−/−*^ mice's spatial memory deficit.

### Reduced reactive astrocyte marker was detected in the hippocampi of *Prdx6*^*−/−*^ mice

**Fig. 7:**
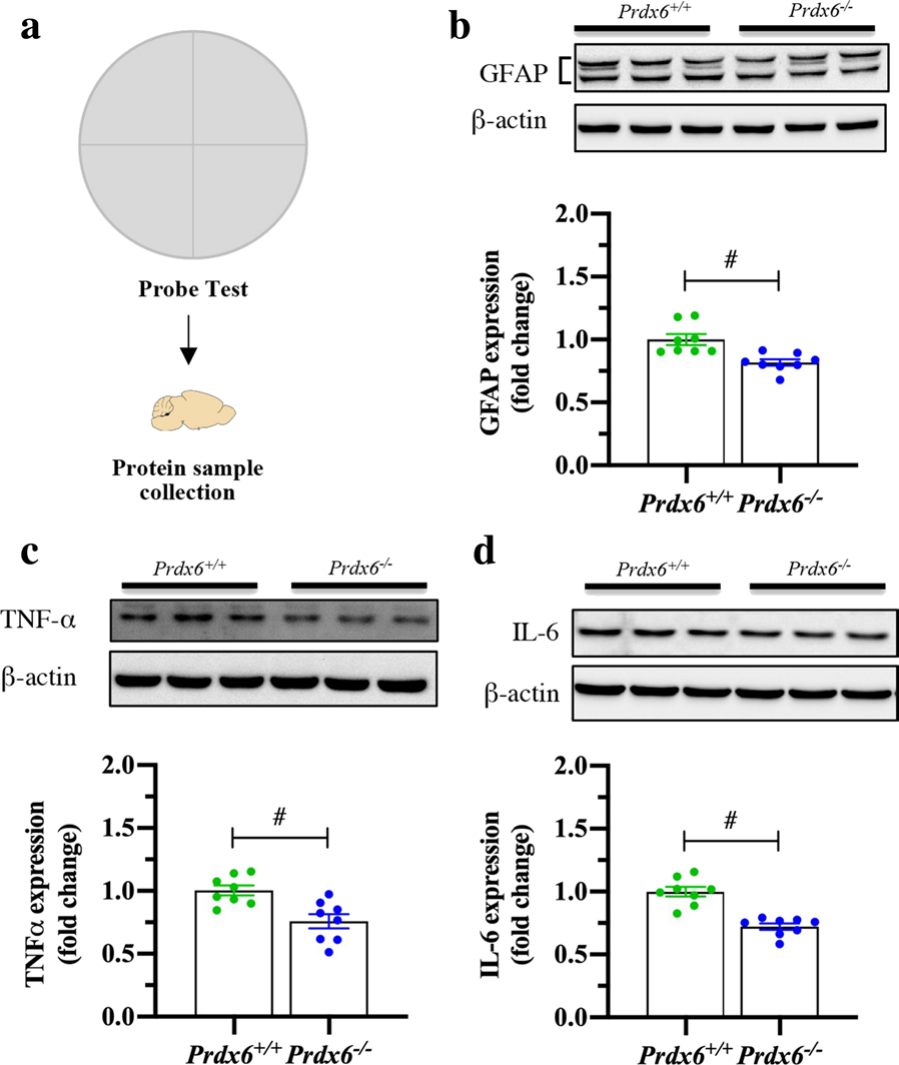
Astrocytic activation and proinflammatory cytokine level in the hippocampus of *Prdx6*^*−/−*^ mice. **a** Schematic of total protein collection from the hippocampus immediately after the probe test. **b**–**d** Representative western blot and quantification of GFAP (**b**), TNFα (**c**), and IL-6 (**d**) in the hippocampus immediately after the probe test (n = 8 mice/group). All data are presented as mean ± SEM. ^#^*p* < 0.05, unpaired Student’s *t*-test following a normal distribution

PRDX6 is highly expressed in the astrocytes, which are the source of cytokine production and release [21]. Previous studies also pointed out a strong relationship between PRDX6 and activation of astrocytes [15]. Here, we proved that PRDX6 was highly expressed in the astrocytes of the hippocampi (Additional file 1: Fig. S4a). We further determined the expression of GFAP (an astrocytic marker), TNFα and IL-6 in the hippocampus. We collected the protein samples immediately after the probe test (Fig. 7a). Analysis of hippocampal protein expression showed that *Prdx6*^*−/−*^ mice had significantly lower GFAP (*t*_14_ = 3.665, *p* = 0.003, Fig. 7b), TNFα (*t*_14_ = 4.565, *p* = 0.000, Fig. 7c) and IL-6 (*t*_14_ = 6.005, *p* = 0.000, Fig. 7d) relative to *Prdx6*^+*/*+^ mice. These findings demonstrated the reduction of astrocyte activation in the hippocampus of *Prdx6*^*−/−*^ mice.

## Discussion

In the present study, we found spatial memory impairment and homeostatic dysregulation of hippocampal long-term potentiation (LTP) in *Prdx6*^*−/−*^ mice. Our results identify the novel role of PRDX6 in spatial memory formation and synaptic plasticity.

A previous report showed that PRDX6 expression is reduced in aged rats with impaired spatial memory [17]. In the present study, we found that *Prdx6*^*−/−*^ mice spent less time within the target quadrant in the probe test (Fig. 1f). MWM performance is affected by stress-induced anxiety-like behaviors and motor function [35, 37]. In this study, *Prdx6*^*−/*−^ mice exhibited higher locomotor activity (Fig. S1), which is consistent with that shown in our recent publication [38]. Although the *Prdx6*^*−/−*^ mice showed higher locomotor activity in open field test, their escape latency to the visible platform (Fig. 1b) and swimming speed in MWM (Fig. 1c) are comparable to their wild-type littermates. Therefore, impaired spatial memory of the *Prdx6*^*−/−*^ mice is attributable to loss of the *Prdx6* gene but not to locomotor function. Our recent report showed that *Prdx6*^*−/−*^ mice exhibited enhanced contextual fear memory, which is also hippocampal-dependent, while their anxiety response evaluated by elevated plus-maze was normal [38]. Many pieces of evidence support that synaptic plasticity in the hippocampus reflects memory function [39, 40], and high-frequency stimulation on Schaffer collateral pathway triggers a persistent enhanced long-term potentiation (LTP) representing long-term memory formation [41]. Our results demonstrated that the spatial memory deficit was correlated with impaired LTP recorded from home-caged *Prdx6*^*−/−*^ mice. The aiPLA2 activity of PRDX6 is necessary for activating NADPH oxidase 2 (NOX2) by acting on its regulatory subunit in endothelial cells and macrophages [42, 43]. NOX2 is also expressed in the hippocampus [44] and affects hippocampal neuronal polarity [45]. Mice lacking regulatory subunits of NOX2 exhibited reduced LTP and impaired spatial memory in a Morris water maze [46]. We thus assume that PRDX6 and NOX2 may participate in the same or related pathway(s) for modulating synaptic plasticity and memory formation. Further investigations are required to delineate their relationship.

The ability to retrieve consolidated memory determines the memory performance during the probe test [47], which is hippocampal-dependent [48]. Reduction of hippocampal LTP is usually correlated with impaired spatial memory [49]. However, some studies report that enhanced LTP is recorded from mice demonstrating defective spatial memory [50–52], indicating LTP and memory performance can be dissociated, as is observed in *Prdx6*^*−/−*^ mice. Taken the impaired probe test performance together with LTP reduction of home-caged *Prdx6*^*−/−*^ mice, and LTP enhancement of trained *Prdx6*^*−/−*^ mice, loss of the *Prdx6* gene can affect synaptic plasticity in the brains differently under naïve and trained conditions. Recent evidence suggests that previous neuronal activation affects the synaptic strength of a given stimulus [53]. In the hippocampus, each pyramidal cell of CA1 subregion receives many inputs, which can potentially increase synaptic plasticity [54]. Thus, the mechanisms to limit total synaptic strength are required to prevent synaptic plasticity saturation, leading to memory impairment [55]. Therefore, we assume that loss of the *Prdx6* gene may affect homeostatic regulation of synaptic strength, as shown in an enhanced hippocampal LTP after a probe test.

Moreover, the hippocampal LTP recorded from trained *Prdx6*^−/−^ mice may be associated with accumulative levels of postsynaptic proteins and downstream signaling molecules [56]. Extracellular signal-regulated protein kinases 1 and 2 (ERK1/2) and its downstream target, cytosolic phospholipase A2 (cPLA2) are known to play crucial roles in synaptic plasticity [57–59], and memory retrieval [60, 61]. The deficit in spatial memory performance of *Prdx6*^*−*/−^ mice can be rescued by suppressing ERK1/2 hyperphosphorylation with MEK inhibitor-U0126, confirming that optimal level of phosphorylated ERK1/2 is important for normal spatial memory retrieval. Our results verify that loss of PRDX6 leads to dysregulation of ERK1/2 phosphorylation, and subsequently causes impaired spatial memory recall and synaptic plasticity regulation.

Postsynaptic density protein 95 (PSD95) is crucial for synaptic plasticity and memory formation [62, 63]. This synaptic protein is known to facilitate ERK1/2 activity [64, 65]. Upregulation of PSD95 in the hippocampus of *Prdx6*^*−/−*^ mice could be the cause of excessive ERK1/2 phosphorylation during memory retrieval. PRDX6 is dominantly expressed in the astrocytes within the hippocampus [66] and regulates their functions [67]. Astrocytes are well known to play a critical role in synaptic plasticity [25], and a decrease in the reactive form of astrocytes is associated with upregulation of PSD95 [68, 69]. Previous reports revealed that lack of PRDX6 causes downregulation of the cytokines such as TNF-α [15] and IL-6 [29], as well as GFAP, a reactive astrocyte marker [67]. Here, we detected downregulation of these molecules, particularly GFAP, indicating decreased reactive astrocytes in the hippocampi of *Prdx6*^*−/−*^ mice. Therefore, we assume that less reactive astrocytes may be correlated with dysregulation of PSD95 and ERK1/2 phosphorylation in the hippocampi, in turn, leading to enhanced LTP and defective spatial memory. Since hippocampal astrocytes play a critical role in synaptic plasticity [25], neurons may try to compensate for the lack of support from astrocytes by elevating expression levels of PSD95 and its downstream targets, pERK1/2 and cPLA2, for maintaining the homeostasis. Although these molecules are upregulated, it did not rescue *Prdx6*^−/−^ mice's spatial memory deficit. More investigations are necessary for understanding the underlying mechanisms. Since PRDX6 plays a critical role in neurogenesis [10], the *Prdx6*^*−/−*^ mice may have unnoted abnormality during development. Therefore, we cannot rule out the possibility that the impaired spatial memory of *Prdx6*^*−/−*^ mice may be related to developmental change.

In summary, our results demonstrate the PRDX6′s role in the regulation of spatial memory and synaptic plasticity through ERK1/2 signaling in the hippocampus. This study helps better understand the molecular mechanism underlying spatial memory-related disorders and suggest PRDX6 as a promising therapeutic target.

## Supplementary Information

**Additional file 1:** *Prdx6*^*−/−*^ mice showed hyperlocomotion in an open field test (Figure S1). Unchanged pro- and mature-BDNF expression in the hippocampus of *Prdx6*^*−/−*^ mice (Figure S2). MEK inhibitor, U0126 significantly decreased pERK1/2 in the hippocampus of *Prdx6*^*−/−*^ mice (Figure S3). And the expression of PRDX6 in hippocampal astrocytes after contextual testing (Figure S4).

**Additional file 2:** The list of specific primers for genotyping (Table S1). The starting point for each trial during visible platform test (Table S2). The start positions for each day during hidden platform training trials (Table S3). The details of antibodies and vector used in this study (Table S4). And the sample sizes of the animals for each experiment (Table S5).

## Data Availability

The data that support the findings of this study are available from the corresponding author upon reasonable request.

## Abbreviations

ACSF: Artificial cerebrospinal fluid
AKT: Protein kinase B
ANOVA: Analysis of variance
BDNF: Brain-derived neurotrophic factor
BSA: Bovine serum albumin
CNS: Central nervous system
CCL5: Chemokine(C–C motif)ligand 5
CAMKII: Ca^2^^+^/calmodulin-dependent protein kinase II
CA1: Cornu ammonis 1
cPLA2: Cytosolic phospholipase A2
ERK: Extracellular signal-regulated kinase
fEPSP: Field excitatory postsynaptic potential
GFAP: Glial fibrillary acidic protein
HT: Heterozygous knockout
HFS: High frequency stimulation
IL-6: Interleukin 6
IL-1β: Interleukin 1β
KO: Knockout
LTP: Long-term potentiation
MEK: Mitogen activated protein kinase
MVM: Morris water maze
PRDX6: Peroxiredoxin6
PCR: Polymerase chain reaction
PVDF: Polyvinylidene fluoride
PSD-95: Postsynaptic density protein 95
PTPδ: Protein tyrosine phosphatase δ
RIPA: Radioimmunoprecipitation assay buffer
SW: Southwest
SDS-PAGE: Sodium dodecyl sulfate polyacrylamide gel electrophoresis
TNF: Tumor necrosis factor
TBST: Tris-buffered saline
NE: Northeast
NOX2: NADPH oxidase 2
