# Supplementary material for: Lack of the peroxiredoxin 6 gene causes impaired spatial memory and abnormal synaptic plasticity
Source: Mol Brain. 2021 Apr 19;14:72. doi: 10.1186/s13041-021-00779-6 (PMC8056661; doi:10.1186/s13041-021-00779-6)
Supplement: Supplementary file 1 — Additional file 1: Prdx6−/− mice showed hyperlocomotion in an open field test (Figure S1). Unchanged pro- and mature-BDNF expression in the hippocampus of Prdx6−/− mice (Figure S2). MEK inhibitor, U0126 significantly decreased pERK1/2 in the hippocampus of Prdx6−/− mice (Figure S3). And the expression of PRDX6 in hippocampal astrocytes after contextual testing (Figure S4). [file 13041_2021_779_MOESM1_ESM.docx]

**Additional Data**

**Lack of the peroxiredoxin 6 gene causes impaired spatial memory and abnormal synaptic plasticity**

Sarayut Phasuk^1,2^, Sureka Jasmin^1^, Tanita Pairojana^1^, Hsueh-Kai Chang^3^, and Ingrid Y. Liu^1, *^


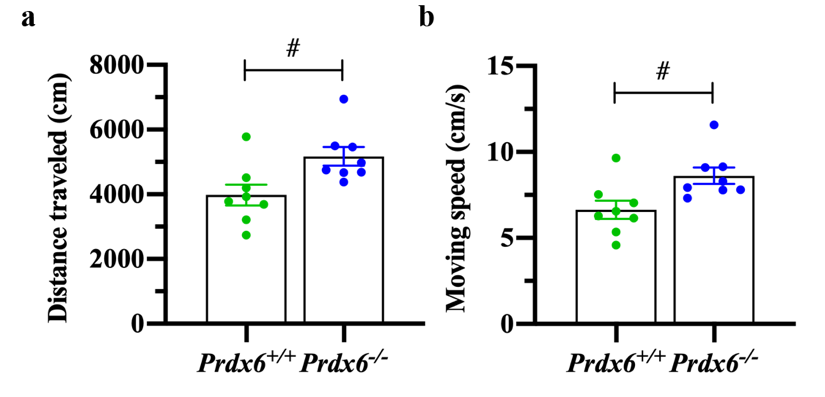


**Additional Data Figure S1.** *Prdx6^-/-^* mice showed hyperlocomotion in an open field test. **(a)** Distance traveled. **(b)** Moving speed. All data are presented as mean±SEM. #*p* < 0.05, unpaired Student’s *t*-test following a normal distribution.


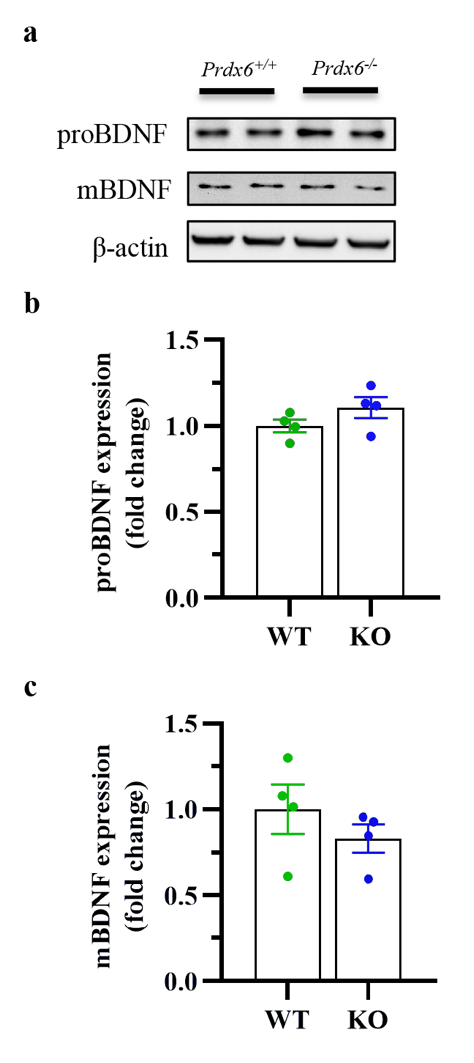


**Additional Data Figure S2.** Unchanged pro- and mature-BDNF expression in the hippocampus of *Prdx6^-/-^* mice. **(a-c)** Representative western blot **(a)** and quantification data of proBDNF **(b)**, and mBDNF **(c)** in the hippocampus immediately after the probe test. All data are presented as mean±SEM.

**
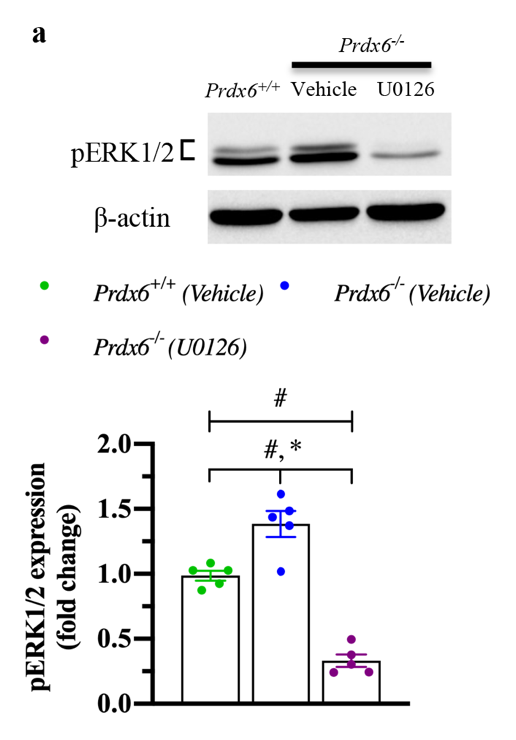
**

**Additional Data Figure S3.** MEK inhibitor, U0126 significantly decreased pERK1/2 in the hippocampus of *Prdx6^-/-^* mice. **(a)** Representative western blot and quantification data of pERK1/2 in the hippocampus collected immediately after the probe test (n = 5 mice/group). All data are presented as mean±SEM. #*p* < 0.05 vs. *Prdx6^+/+^* (Vehicle) and **p* < 0.05 vs. *Prdx6^-/-^* (Vehicle), one-way ANOVA followed by Bonferroni’s post hoc test.

**
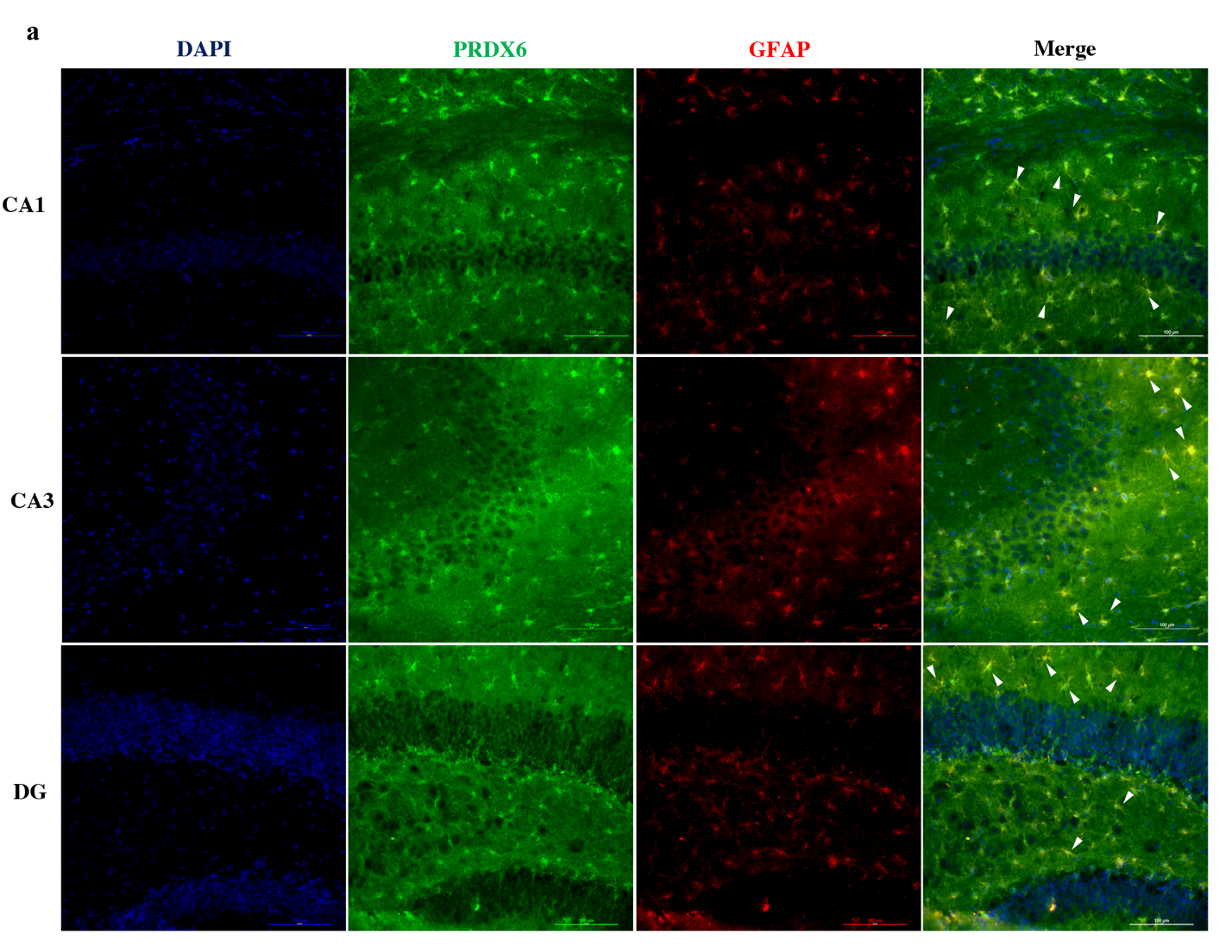
Additional Data Figure S4.** The expression of PRDX6 in hippocampal astrocytes after contextual testing. **(a)** Immunofluorescent images of brain sections from *Prdx6^+/+^* mice after contextual testing. The merged images of double staining for PRDX6 (green) and GFAP (red) showing the colocalization (yellow, white arrows) of PRDX6 with GFAP in the CA1, CA3 and DG subregions of the hippocampus. PRDX6, peroxiredoxin 6; GFAP, glial fibrillary protein; CA1, cornu ammonis 1; CA3, cornu ammonis 3; DG, dentate gyrus.
